# Sequential Shifting in T-helper and T-cytotoxic Subset Cell Population in Mild and Severe COVID-19 Patients Infected With Variant B.1.61

**DOI:** 10.7759/cureus.40556

**Published:** 2023-06-17

**Authors:** Jyotsna Agarwal, Namrata P Awasthi, Shivani Singh, Vandana Tiwari

**Affiliations:** 1 Microbiology, Dr. Ram Manohar Lohia Institute of Medical Sciences, Lucknow, IND; 2 Pathology, Dr. Ram Manohar Lohia Institute of Medical Sciences, Lucknow, IND; 3 Biochemistry, Dr. Ram Manohar Lohia Institute of Medical Sciences, Lucknow, IND

**Keywords:** disease severity, flowcytometry, t-cytotoxic cells, t-helper cells, covid-19

## Abstract

Aim: Severe acute respiratory syndrome coronavirus-2 (SARS-CoV-2) modulates antiviral immunity via T cells, but whether these cells are active or abundant in coronavirus disease 2019 (COVID-19) patients is unknown. The present study aimed to investigate the temporal shifting in the T-cell population and their subsets, T-Helper (Th) cell (CD4) and T-Cytotoxic (Tc) cell (CD8) in COVID-19 patients.

Method: Thirty confirmed COVID-19 patients (nasal swab reverse transcription-polymerase chain reaction (RT-PCR) confirmed) were enrolled. On the basis of oxygen saturation (SpO2) levels, patients were stratified into two categories: (i) mild (n=11) having fever and SpO2 level >95%, and (ii) severe (n=19) on the ventilator, and in the intensive care unit (ICU) as per the Indian Council of Medical Research (ICMR) guidelines. Thirty age-sex-matched controls without infectious diseases unrelated to COVID-19 were also enrolled in the study. Patients with inflammatory diseases and severe comorbidities that compromise immunity were excluded from the study. Immunophenotyping flow cytometry assay was used to evaluate T-cell viability, Th, and Tc cells population in mild and severe COVID-19 patients on day 1 (at admission) and day 4 (decreasing the infection load) in the second COVID-19 wave (variant: B.1.61).

Categorical variables were expressed as frequency and percentage and p-values were calculated by Chi-square test. All the variables were represented in median and Q1 (25 percentile) and Q3 (75 percentile). The Mann-Whitney test was used to compare the study groups. The Δ mean differences were calculated by using the Paired samples t-test. The statistically significant level was taken as p<0.05.

Results: Hemoglobin, total leukocyte count (TLC), lymphocytes, monocytes, and eosinophils were significantly reduced in patients (p<0.05). A significant decrease of CD4 and CD8 cells in severe COVID-19 patients vs. controls (CD4, median 49; CD8, 40.12; p>0.05) was seen. Th-EM (effector memory)-Tim-3 (T-cell immunoglobulin domain and mucin domain 3)+ was significantly higher (p=0.002) however, Tc-EMRA (effector memory cells re-expressing)-Tim-3+, Tc-Naive-Tim-3+, Tc-EM-PD1+ and Tc-CM (central memory)-Tim-3+ significantly reduced (p<0.05) in mild COVID-19 patients than controls. Similarly, in severe COVID-19 patients, Th-EMRA-Tim-3+, Th-Naive-PD1+, Th-EM-PD1+, Th-EM-Tim 3+ and Th-CM-Tim-3+ showed a significant reduction (p<0.05) and Tc-EMRA-Tim-3+, Tc-Naive-Tim-3+, Tc-EM-PD1+, and Tc-CM-Tim-3+ showed similar results.

In mild vs. severe group, decreased T-cells (p=0.001), Th-EMRA-Tim-3+ (p=0.024), and Th-Navie-Tim-3+ (p=0.005), and significantly increased (p<0.05) Tc-Naive-Tim3+ (p=0.001), Tc-EM-Tim-3+ (p=0.031), and Tc-CM-Tim-3+ (p=0.08) were observed. Severe COVID-19 patients showed a significant increase in Th-Naive-Tim3+ (day 4-day 1; δ43, p=0.019), Th-EM-Tim3+ (δ 16.24, p=0.033), and Th-CM-Tim3+ (δ 13.57, p=0.041).

Conclusion: T-cell populations and CD8 subset help to differentiate the mild and severe COVID-19 patients. Monitoring T cells, especially CD8 subset changes, has important implications for diagnosing and treating mild and severe patients being critically ill.

## Introduction

The coronavirus disease 2019 (COVID-19) pandemic caused by severe acute respiratory syndrome coronavirus 2 (SARS-CoV-2) has resulted in nearly 800 million confirmed cases and seven million deaths worldwide as of May 2023 [1,2].

Even amid an active infection, the immune system invests in the host’s future by selecting activated T cells to become memory progenitors. If the primary response successfully wards off the infection, the organism will, in most cases, go on to preserve part of that immune response in the form of memory T cells. Because of the successful resolution of the prior infection, these memory T cells are a known quantity and are thus preserved at an increased frequency throughout the body, ready to mediate an enhanced and accelerated response to reinfection [3]. However, T-cell memory is not only important as a source of new effector T cells; memory Th responses can enhance memory B cell responses upon rechallenge, which becomes particularly important in the context of pathogen evolution to evade antibody recognition. For these reasons, the major goal of all vaccines against SARS-CoV-2 should be to elicit T-cell memory in both the circulation and tissues. In the context of both natural SARS-CoV-2 infection and vaccination, tracking the stability of circulating and tissue-resident T-cell memory over months and years in humans and animal models will be crucial.

Although work on SARS-CoV-2 suggests that T-cell memory to SARS-CoV-2 is likely long-lived, further research and more time are required to assess the immunity to SARS-CoV-2 fully [4]. Furthermore, whether pre-existing SARS-CoV-2-reactive T cells in naive individuals provide beneficial immunity, promote an ineffective response by biasing the responding population, or cause immunopathology remains unanswered, but these populations will likely have a role in the development of anti-SARS-CoV-2 memory response.

T cells have a pivotal role in antiviral immunity. Effector T cells eradicate virus-infected cells, contribute to the innate antiviral response, and promote B cell responses, culminating in the generation of virus-specific antibodies [5]. Conventional T cells, defined by the expression of the cell-surface receptors CD4 and CD8, are characterized by their expression.

CD8 cells can recognize SARS-CoV-2 antigen in a proportion of healthy individuals and COVID-19 patients [6-8]. Furthermore, CD8 cells frequently display depleted characteristics in this disease and drastically reduce cell numbers in certain severe patients [9]. This raises questions about the inability of CD8T cells to mediate cellular protection during the peak of the infection [10].

Similar to CD8 cells, evidence is that CD4 cells exhibit functional impairment and elevated expression of activation and/or exhaustion markers in COVID-19 patients [11]. Human peripheral CD4+ T cells can be characterized as naive (CCR7+CD45RA+), central memory (CCR7+CD45RA-), and effector-memory (CCR7-, CD45RA-) cells that respond differently during antigen re-exposure [12].

Most acute viral infections, including SARS-CoV-2 infection in humans, activate and alter T cells. Hence, quantitative and/or qualitative shifts in the T cells and their subsets (CD4 and CD8 cells) have been associated with SARS-CoV-2 infection [13]. Various studies have observed an increase in the frequency of activated T cell phenotypes and the expression of T cell exhaustion-related surface markers, such as programmed cell death 1 (PD-1) and T-cell immunoglobulin mucin domain 3 (TIM3) [14]. COVID-19 is highly contagious and its pathologic mechanism has attracted much attention [2,15]. Most patients recovered after careful treatment, but some developed severe and critical illnesses [16]. The detailed mechanisms underlying severe respiratory syndrome caused by the COVID-19 coronavirus remain unclear [14]. There is no effective treatment to rescue severe patients who might be turned into patients with critical illnesses [15]. Therefore, clarifying the pathological differences between moderate, severe, and critical patients is urgent. Thus, the current research aimed to identify the status of T-cell subsets (CD4 and CD8) surface markers status (populations) to differentiate disease severity and dynamic change in the cell population at day 1 (infection) and day 4 (viral load).

## Materials and methods

Study population

In the current study, 60 subjects were enrolled during the second wave of COVID-19 (variant B.1.61), which included 30 confirmed COVID-19 patients (nasal swab reverse transcription-polymerase chain reaction (RT-PCR) confirmed) admitted to the COVID-19 ward at Dr. Ram Manohar Lohia Institute of Medical Sciences, Lucknow, India, and 30 normal healthy subjects (age-sex matched with cases) enrolled as the control group in the study. Furthermore, on the bases of oxygen saturation (SpO2) levels, the 30 COVID-19 patients were stratified into two categories: (i) mild (n=11) with fever and SpO2 level >95%, and (ii) severe (n=19) on the ventilator and in intensive care unit (ICU) are required as per the Indian Council of Medical Research (ICMR) guidelines. COVID-19 patients with mild diabetes and hypertension as comorbidity were included in the study. The exclusion criteria were: age <18 years of age, pregnant women, other lung disease, renal disease, malignancy, inflammatory diseases, and severe comorbidities that compromised immunity.

Blood samples were taken at the time of admission (day 1) and then on day 4, irrespective of disease progression or recovery in clinical symptoms, lab results, or arterial oxygen saturation after obtaining consent from the patient’s guardian/attendant. The ethical approval was obtained from the Institutional Ethics Committee of Dr. Ram Manohar Lohia Institute of Medical Sciences (approval number: IEC-113/20).

Reagents and panel

For the immunophenotyping study (total T cells, viable T cell, and T-cell subset analysis), the following fluorescently labeled anti-human monoclonal antibodies with fluorochrome and viability dye were used from BD Biosciences (San Jose, California, United States): anti-CD3 V500c, CD4 APC- R700, CD8 Per CP, CD197 PE (2LI-A), CD45RA FITC (HI100), CD279 BV605 (EH12.1), CD366 Alexa647 (TD3), CD25 BV421 (MA251), CD127 PE-Cy7 (HIL-TRMZ1), and fixable viability stain (FVS) 780 in both the samples of each enrolled study subject for measurements by flow cytometry.

Laboratory investigations

Hematological Parameters

Complete blood count was analyzed using five-part white blood cells (WBC) differential using advanced multi-angle polarized scattered separation (MAPSS) technology (Abbott Laboratories, Chicago, Illinois, United States).

T Cell and Subsets Analysis by Flowcytometry

Peripheral blood samples were collected in ethylenediaminetetraacetic acid (EDTA) vacutainer vials on days 1 and day 4 and mononuclear cells were separated. In brief, 300 μL of blood was mixed with 700 μL of sheath fluid and centrifuged at 300xg for five minutes. The supernatant was discarded, the cell pellet was resuspended in 100 μL of sheath fluid, 1 μL of FVS dye was added, and incubated for 15 minutes in the dark at room temperature. The cell suspension was washed twice with 2 ml of stain buffer by centrifugation at 300xg for five minutes. Finally, the cell pellet was resuspended into 100 μl stain buffer, and 50 μL brilliant stain buffer was added after that; antibodies were added to the cell suspension and vortexed. The tube was incubated for 20 minutes in the dark at room temperature, followed by adding 2 mL of 1X FACS lyse solution in each tube, vortexed, and incubated for 10-12 minutes in the dark at room temperature. The cell suspension was further centrifuged at 250 g for five minutes. The supernatant was discarded carefully, and the pellet was broken. Again 2 mL sheath fluid was added, and the cell suspension was centrifuged at 300 g for five minutes.

The final cell pellet was resuspended in a 300 μL sheath to acquire stained cells in BD FACSCanto™ flow cytometer (Becton, Dickinson and Company, Franklin Lakes, New Jersey, United States) with a BVR (blue-violet-red) laser.

Gating Strategy

Singlet gating was used to exclude doublets. Further, sequential gating on forward and side scatter followed by CD3-based gating was applied to study CD4 and CD8 T cells and their subsets. One million cells were acquired and analyzed using BD FACSDiva™ Software (Becton, Dickinson and Company).

Statistical analysis

Categorical variables were expressed as frequency and percentage and the p-values were calculated by Chi-square test. All the variables were represented in Median and Q1 (25 percentile) and Q3 (75 percentile). The Mann-Whitney test was used to compare the study groups. The Δ mean differences were calculated by using the paired samples t-test. The statistically significant level was taken as p<0.05. All data were analyzed by using IBM SPSS Statistics for Windows, Version 21.0 (Released 2012; IBM Corp., Armonk, New York, United States).

## Results

General and hematological characteristics of study population

The baseline characteristics of the study groups are listed in Table 1. Patients had symptoms like cough, fever, sore throat, rhinorrhea, shortness of breath, and comorbidities. There was no significant difference between the age and sex of the mild and severe groups of the COVID-19 patients (p=0.109 and 0.416, respectively).

**Table 1:** General characteristics of COVID-19 patients Applied Chi-square test/Fisher exact test as appropriate COVID-19: coronavirus disease 2019

Variables		Category		p-value
		Mild, n (%)	Severe, n (%)	
Age (Years)	31-45	2 (18.2)	9 (47.4)	0.109
	46-66	9 (81.8)	10 (52.6)	
	Mean±SD	54.3 1±14.66	48.85±12.40	0.242
Sex	Male	8 (72.7)	11 (57.9)	0.416
	Female	3 (27.3)	8 (42.1)	
Cough	Yes	6 (54.5)	11 (57.9)	0.858
	No	5 (45.5)	8 (42.1)	
Fever	Yes	7 (63.6)	10 (52.6)	0.557
	No	4 (36.4)	9 (47.4)	
Sore Throat	Yes	8 (72.7)	19 (100)	NA
	No	3 (27.3)	0	
Rhinorrhea	Yes	6 (54.5)	11 (57.9)	0.85
	No	5 (45.5)	8 (42.1)	
Shortness of Breath	Yes	5 (45.5)	12 (63.2)	0.260
	No	6 (54.5)	7(36.8)	
Co-morbidities	Yes	8 (72.7)	15 (78.9)	0.69
	No	3 (27.3)	4 (21.1)	

Table 2 represents the hematological parameters in which lymphocytes, monocytes, and eosinophils were significantly decreased (p<0.05); in contrast, neutrophils and platelets were significantly increased (p<0.05) in COVID-19 patients compared to the control group.

**Table 2:** Comparison of Hematological Parameters in COVID-19 Patients and Controls. Data were represented in Median, lower Quartile (Q1) and upper Quartile (Q3).; the Mann-Whitney U test was used to calculate the p-value. *p-value <0.05 considered as statistically significant. Hb: hemoglobin; Hct: hematocrit; RBCs: red blood cells; MCV: mean corpuscular volume; MCH: mean corpuscular hemoglobin; MCHC: mean corpuscular hemoglobin concentration; WBC: white blood cells; COVID-19: coronavirus diseae 2019

Variables	COVID-19 Patients (N=30), Median (Q1-Q3)	Controls (N=30), Median (Q1-Q3)	p-value
Hb (g/dL)	11.90 (9.10-12.90)	12.70 (11.95-13.10)	0.124
Hct (%)	35.50 (29.10-38.90)	38.75 (35.30-40.50)	0.082
RBCs (×10^6^/uL)	4.16 (3.49-4.67)	4.29 (3.84-4.66)	0.684
MCV (fL)	87.70 (78.70-92.80)	88.35 (85.65-94.05)	0.268
MCH (pg)	28.00 (26.80-31.00)	28.90 (28.00-31.75)	0.075
MCHC (%)	33.30 (31.80-34.00)	32.45 (32.10-33.20)	0.871
WBC count (× 10^3^/uL)	9.50 (7.00-12.30)	7.23 (6.56-9.41)	0.075
Neutrophil (%)	85.00 (77.00-86.00)	58.00 (52.00-66.00)	<0.0001*
Lymphocyte (%)	11.00 (8.00-19.00)	28.50 (21.50-35.50)	<0.0001*
Monocyte (%)	2.00 (1.00-4.00)	7.00 (6.00-10.00)	0.001*
Eosinophil (%)	2.00 (1.00-2.00)	3.00 (2.00-4.00)	<0.0001*
Platelet count (×10^3^/uL)	228.00 (160.00-289.00)	155.50 (129.00-199.00)	0.023*

**Figure 1:**
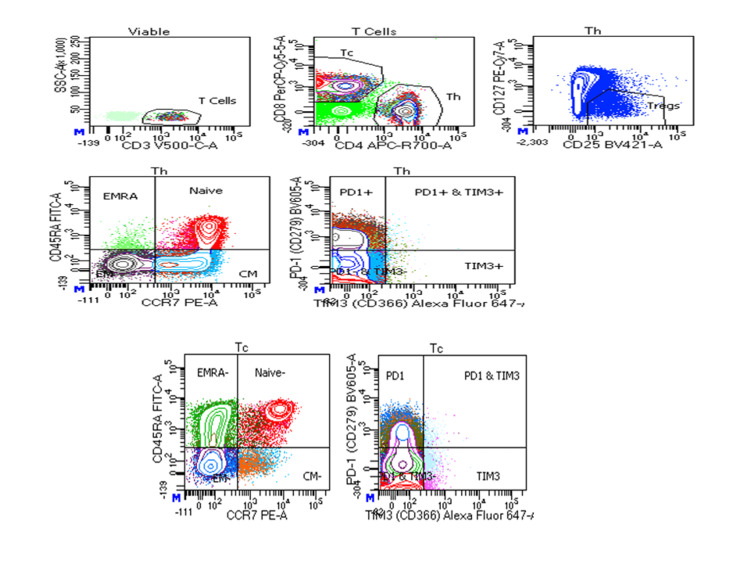
Flow cytometric analysis of T cells (Th and Tc) viability and their subsets in controls Th: T-helper; Tc: T-cytotoxic; COVID-19: coronavirus disease 2019; EMRA: effector memory cells re-expressing; PD1: programmed cell death protein 1; TIM3: T-cell immunoglobulin mucin domain 3

**Figure 2:**
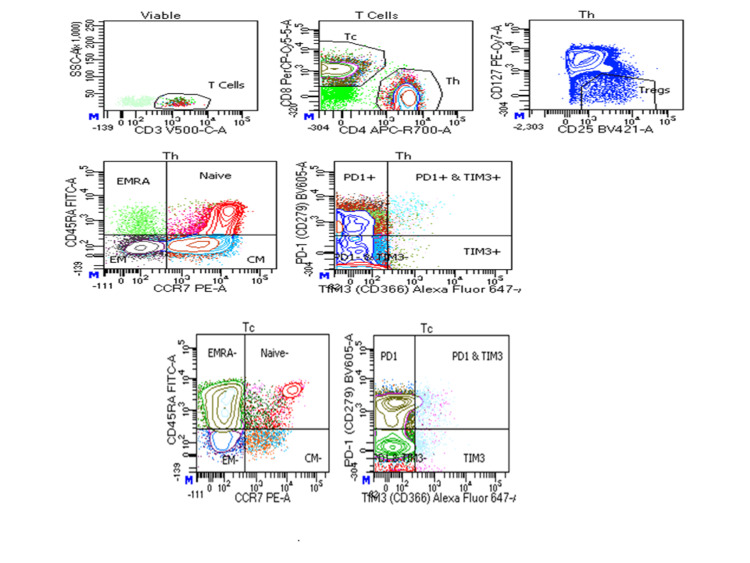
Flow cytometric analysis of T cells (Th and Tc) and their subsets in mild COVID-19 patients Th: T-helper; Tc: T-cytotoxic; COVID-19: coronavirus disease 2019; EMRA: effector memory cells re-expressing; PD1: programmed cell death protein 1; TIM3: T-cell immunoglobulin mucin domain 3

**Figure 3:**
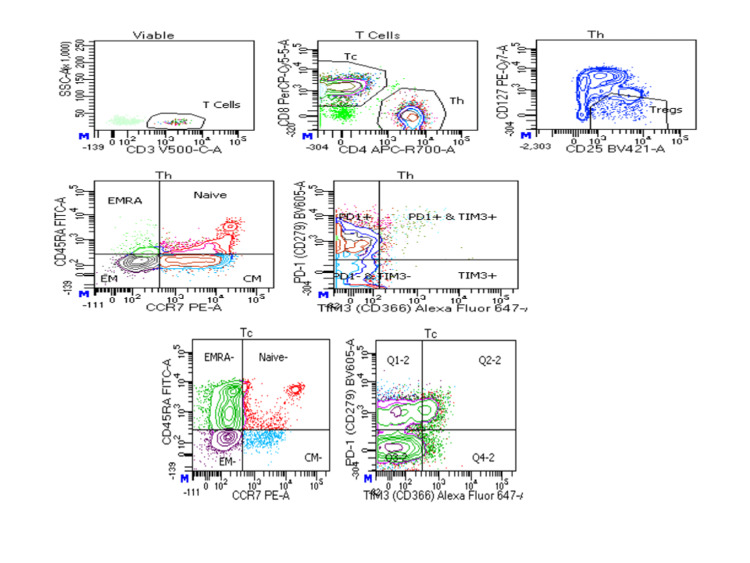
Flow cytometric analysis of T cells (Th and Tc) and their subsets in severe COVID-19 patients Th: T-helper; Tc: T-cytotoxic; COVID-19: coronavirus disease 2019; EMRA: effector memory cells re-expressing; PD1: programmed cell death protein 1; TIM3: T-cell immunoglobulin mucin domain 3

**Figure 4:**
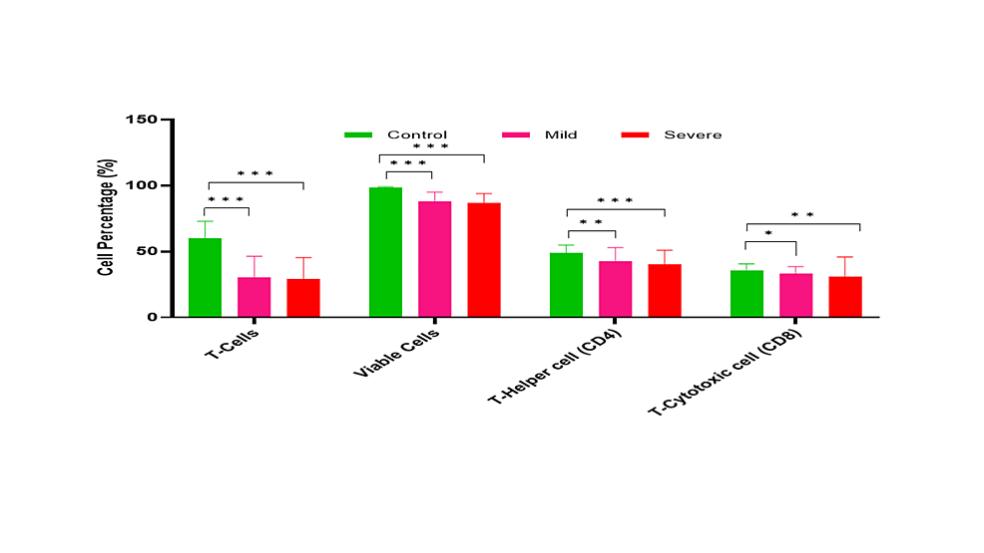
Comparative analysis of T cells, viability, and their subsets (Th and Tc) in controls, and mild and severe COVID-19 patients. *- p-value <0.05, **- p-value<0.01, ***- p-value<0.001 Th: T-helper; Tc: T-cytotoxic; COVID-19: coronavirus disease 2019

The overall T-cell viability was reduced in severe COVID-19 patients followed by mild COVID-19 patients as compared to the control (p<0.0001) (Figures 1-4).

Status of Th (CD4) and Tc (CD8) subset cells in mild COVID-19 patients

**Figure 5:**
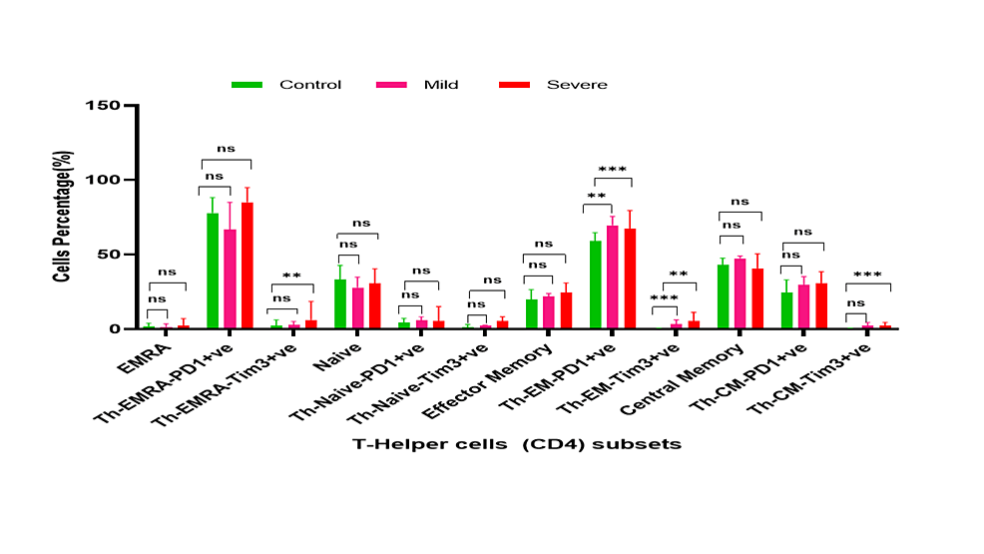
Representative graph of T-helper cells and their subsets in mild and severe cases of COVID-19 and controls. *- p-value <0.05, **- p-value<0.01, ***- p-value<0.001* and ns-non significant. Th: T-helper; Tc: T-cytotoxic; COVID-19: coronavirus disease 2019; EMRA: effector memory cells re-expressing; PD1: programmed cell death protein 1; TIM3: T-cell immunoglobulin mucin domain 3; CM: central memory; EM: effector memory

**Figure 6:**
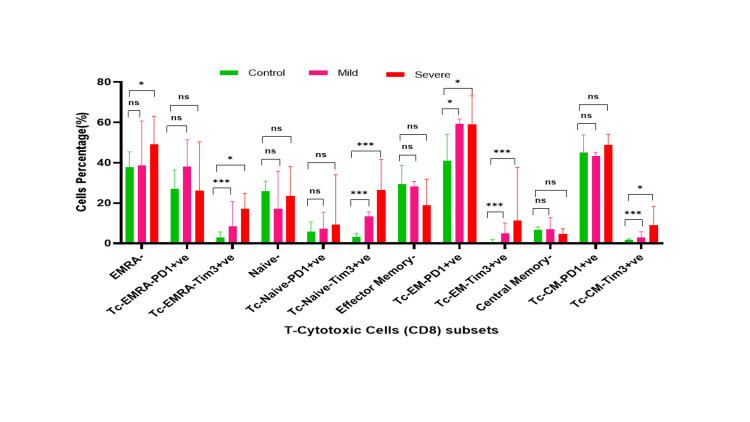
Representative graph of T-cytotoxic cells and their subsets in mild and severe cases of COVID-19, and controls. *- p-value <0.05, **- p-value<0.01, ***- p-value<0.001 Th: T-helper; Tc: T-cytotoxic; COVID-19: coronavirus disease 2019; EMRA: effector memory cells re-expressing; PD1: programmed cell death protein 1; TIM3: T-cell immunoglobulin mucin domain 3; CM: central memory; EM: effector memory

In mild COVID-19 patients' CD4 subset, Th EM-Tim-3+ (Median 3.3; Q1-Q3 2.45-5.6.3) population was significantly higher as compared to controls (Median 0.5; Q1-Q3 0.3-0.85; p=0.002). However, CD8 subsets; Tc EMRA (effector memory cells re-expressing)-Tim-3+ (Median 8.4; Q1-Q3-4 25-20.75 vs. Median 2.75; Q1-Q3 1.1-5.65, p=0.003), Tc Naive-Tim-3+ (Median 13.4; Q1-Q3 -7.8-15.55 vs. Median 3.3; Q1-Q3 -2.3-38.75; p<0.0001), Tc EM-PD1+ (Median 59.2; Q1-Q3 44.65-61.6 vs. Median 40.9; Q1-Q3 -26.2-54.1; p=0.036) and Tc-CM-Tim-3+(Median 3.0; Q1-Q3 -2.4-5.7 vs. Median 1.6; Q1-Q3 1.1-2.25; p=0.002) showed a significant increase as compared to controls (Figures 1-3, 5, 6).

Status of Th (CD4) and Tc (CD8) subset cells in severe COVID-19 patients

The CD4 subset including Th EMRA-Tim-3+ (Median 6.2; Q1-Q3 -3.7-18.6 vs. Median 2.35; Q1-Q3 0.65-6.15; p=0.004), Th Naive-PD1+ (Median 5.45; Q1-Q3 -1.9-152 vs Median 4.4; Q1-Q3 2.4-7.2; p<0.0001), Th-EM (effector memory)-PD1+ (Median 67.45; Q1-Q3 -59.2-79.6 vs. Median 59.15; Q1-Q3 45.1-64.8; p<0.0001), Th EM-Tim 3+ (Median 5.65; Q1-Q3 -4.0-11.3 vs. Median 0.5; Q1-Q3 0.3-0.85; p=0.009), and Th CM (central memory)-Tim-3+ (Median 2.4; Q1-Q3 -1.3-4.5 vs. Median 0.5; Q1-Q3 -0.3-0.85; p<0.0001) showed a significantly higher in severe COVID-19 patients as compared to control. Similarly, CD8 subset cells including Tc EMRA-Tim-3+ (Median 17.2; Q1-Q3 -10.5-24.8 vs. Median 2.75; Q1-Q3 1.1-5.65; p<0.0001), Tc Naive-Tim-3+ (Median 26.4; Q1-Q3 -20.3-41.6 vs. Median 3.3; Q1-Q3 -2.3-4.95; p<0.0001), Tc EM-PD1+ (Median 58.85; Q1-Q3 -44.3-73.2 vs. Median 40.9; Q1-Q3 -26.2-54.1; p=0.011) and Tc CM-Tim-3+ (Median 9.0; Q1-Q3-5.7-18.3 vs. Median 1.6; Q1-Q3 1.1-2.25; p<0.0001) showed a significant increment as compared to controls (Figures 5-6).

Patterns of Th (CD4) and Tc (CD8) subset cells in mild and severe COVID-19 patients

The T cell population was significantly decreased in COVID-19 patients (Severe: Median 30; Q1-Q3 46.4-18.5 vs. mild Median: 35.7; Q1-Q3 -40.8-21.35; p=0.001). The CD4 subsets were Th EMRA-Tim-3+ (Median 6.2; Q1-Q3 -3.7-18.6 vs. Median 2.8; Q1-Q3 -1.5-5.15; p=0.024), and Th Navie-Tim-3+ (Median 5.35; Q1-Q3 -3.5-8.4 vs. Median 2.5; Q1-Q3 -1.95-2.65; p=0.005). Severe COVID-19 patients demonstrated a significant decrease compared to the mild group. Similarly, the CD8 subsets Tc Naive-Tim3+ (Median 26.4; Q1-Q3 20.3-41.6 vs.Median 13.4; Q1-Q3 -7.8-15.5; p=0.001), Tc EM-Tim-3 + (Median 11.3; Q1-Q3 -6.2-37.8 vs. Median 4.9; Q1-Q3 3.65-10.1; p=0.031) and Tc CM-Tim-3+ (Median 9.0; Q1-Q3 -5.7-18.3 vs. Median 3.0; Q1-Q3 2.4-5.7; p=0.08) were significantly increased in severe patients as compared to mild patients (p<0.05). However, other CD4 and CD8 subsets did not show significant differences between the mild and severe groups (Figures 5-6).

Dynamic changes (day 1 and day 4) in Th (CD4) subset cells in mild and severe COVID-19 patients

**Figure 7:**
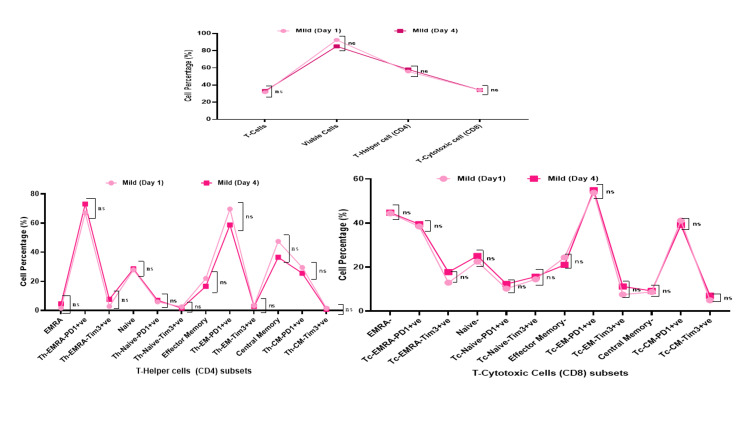
Relationship between the T cells, T cell viability, Th (CD4) cell, Tc (CD8) cell, and their subsets in Mild COVID-19 patients on day 1 and day 4. *- p-value <0.05, **- p-value<0.01, ***- p-value<0.001 Th: T-helper; Tc: T-cytotoxic; COVID-19: coronavirus disease 2019; EMRA: effector memory cells re-expressing; PD1: programmed cell death protein 1; TIM3: T-cell immunoglobulin mucin domain 3; CM: central memory; EM: effector memory; ns: not significant

**Figure 8:**
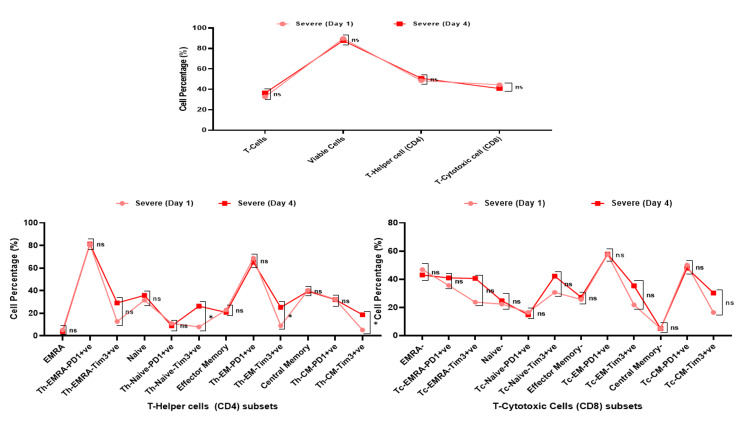
Relationship between the Tcells, T-cell viability, Th (CD4) cells, Tc (CD8) cells, and their subsets in severe COVID-19 patients on day 1 and day 4. *- p-value <0.05, **- p-value<0.01, ***- p-value<0.001 Th: T-helper; Tc: T-cytotoxic; COVID-19: coronavirus disease 2019; EMRA: effector memory cells re-expressing; PD1: programmed cell death protein 1; TIM3: T-cell immunoglobulin mucin domain 3; CM: central memory; EM: effector memory; ns: not significant

There was no significant difference in CD4 and CD8 subsets cell populations on day 1 and day 4 in the mild group of COVID-19 patients (Figure 7). While severe COVID-19 patients showed a significant increase in Th-Naive-Tim3+ (day 4: Mean±SD 26.30±31.60- 7.87±8.94; day1: Δ18.43; p=0.019), Th-EM-Tim3+ (day 4: Mean±SD 25.21±30.68-8.97±8.90; day 1: Δ16.24; p=0.033), and Th-CM-Tim3+ (day4: Mean±SD 18.70±26.22-5.13±9.63; day 1: Δ 13.57; p=0.041) (Table 3, Figure 8).

**Table 3:** Comparision between day 1 and day 4 altered flow cytometry profiling in COVID-19 patients based on severity. Paired t-test were used to calculate the difference between Day 1 and Day 4 *p-value <0.05 is considered as statistically significant. EMRA: effector memory cells re-expressing, PD1: programmed cell death protein 1; Th: T-helper; Tc: T-cytotoxic; COVID-19: coronavirus disease 2019; TIM3: T-cell immunoglobulin mucin domain 3; ns: not significant

Categories	Variables	Δ Mean Difference	p-value
Mild	T Cells	1.44	0.861
	Viable Cells	-7.46	0.111
	Th cell (CD4)	2.35	0.687
	EMRA	0.93	0.809
	Th-EMRA-PD1+	-0.32	0.963
	Th-EMRA-Tim3+	3.60	0.293
	Naive	5.83	0.387
	Th-Naive-PD1+	3.45	0.156
	Th-Naive-Tim3+	2.37	0.472
	Effector Memory	-5.29	0.175
	Th-EM-PD1+	-7.32	0.177
	Th-EM-Tim3+	1.00	0.675
	Central Memory	-1.50	0.778
	Th-CM-PD1+ve	-4.55	0.116
	Th-CM-Tim3+ve	-0.57	0.701
	T-Cytotoxic cell (CD8)	-0.01	0.998
	EMRA-	0.31	0.974
	Tc-EMRA-PD1+	1.03	0.878
	Tc-EMRA-Tim3+	4.79	0.428
	Naive-	2.57	0.706
	Tc-Naive-PD1+	2.10	0.561
	Tc-Naive-Tim3+	1.17	0.809
	Effector Memory	-3.37	0.518
	Tc-EM-PD1+	1.11	0.817
	Tc-EM-Tim3+	3.67	0.375
	Central Memory-	0.50	0.838
	Tc-CM-PD1+	-2.00	0.536
	Tc-CM-Tim3+	2.12	0.567
Severe	T-Cells	3.78	0.578
	Viable Cells	-2.18	0.308
	T-Helper cell (CD4)	2.29	0.605
	EMRA	-1.28	0.516
	Th-EMRA-PD1+	0.18	0.974
	Th-EMRA-Tim3+	16.50	0.058
	Naive	4.29	0.468
	Th-Naive-PD1+	-2.21	0.570
	Th-Naive-Tim3+	18.43	0.019*
	Effector Memory	-2.15	0.550
	Th-EM-PD1+	-3.67	0.437
	Th-EM-Tim3+	16.24	0.033*
	Central Memory	-0.85	0.862
	Th-CM-PD1+	-0.19	0.953
	Th-CM-Tim3+	13.57	0.041*
	T-Cytotoxic cell (CD8)	-3.58	0.447
	EMRA-	-3.92	0.468
	Tc-EMRA-PD1+	5.32	0.514
	Tc-EMRA-Tim3+	16.84	0.079
	Naive-	2.32	0.690
	Tc-Naive-PD1+	-1.75	0.701
	Tc-Naive-Tim3+	11.47	0.165
	Effector Memory	1.60	0.793
	Tc-EM-PD1+	0.19	0.976
	Tc-EM-Tim3+	13.53	0.158
	Central Memory	-0.01	0.992
	Tc-CM-PD1+	-2.17	0.633
	Tc-CM-Tim3+	13.88	0.147

## Discussion

The present pilot study analyzed the immune cells population in mild and severe COVID-19 patients and compared them with normal controls. The lymphocytes, monocytes, and eosinophils were significantly decreased in severe patients as compared to mild patients. In contrast, neutrophils were increased in both groups of COVID-19 patients. The present study concluded that thrombocytopenia and lymphopenia may indicate COVID-19 disease. The effects of viral pneumonia on the immune system include decreasing leukocyte and lymphocyte counts [16,17].

Our results demonstrated that T cells, viable cells, T regulatory cells, Th (CD4) cells, and Tc (CD8) cells were significantly reduced in severe patients as compared to mild. There are two layers of adaptive antiviral responses from an immunological perspective. First, CD8 T cell response is initially programmed to prevent the disease from progressing to a severe phase. Second, Th cells are poised to stimulate and program B cells to generate neutralizing antibodies against specific antigens [18], conferring durable humoral immunity [19]. Th cells (CD4) are crucial for adaptive immunological responses [20]. Th (CD4) cells may develop into various subsets in response to antigen presentation [21].

Moreover, Tc (CD8) cells restricted by class I major histocompatibility complex molecules are essential for developing immunity to the influenza virus because they identify internal viral proteins that are conserved across diverse viral strains [22,23]. The novel coronavirus is highly comparable to SARS and Middle East Respiratory Syndrome (MERS); both severe respiratory viruses are controlled by CD4 and CD8 [24]. However, modest CD4 and CD8 reductions have been documented in COVID-19 patients [7,25].

Furthermore, the present investigation endeavors to elucidate unresolved inquiries concerning the memory of CD8+ T cells in response to SARS-CoV-2 infection in humans through tracking individual memory CD8+ T cell clones. Similar to previous research, our study observed a noteworthy reduction in CD4 and CD8 cells among severe patients compared to controls [10,26].

The categorization of immune CD4+ and CD8+ T cells into four primary subsets is determined by the surface expression of CCR7 and CD45RA. The entities mentioned above exhibit varying degrees of maturation and differentiation of T cells, which are characterized by unique functional properties. The various subsets that are present include the naïve CD4+ T cell subsets (CCR7+CD45RA+), central memory CD4+ T cells (CCR7+CD45RA−), effector memory CD4+ T cells (CD45RA+), revertant effector memory cells (CCR−CD45RA+) (T_EMRA_) [27,28]. The current investigation involved an analysis of various CD4 and CD8 subsets, including Th EMRA-Tim-3+, Th Navie-Tim-3+, Tc Naïve-Tim3+, Tc EM-Tim-3+, and Tc CM- Tim-3+. Furthermore, it has been reported that CD27-CD28-T cells possess a high level of effector functionality that is comparable to that of the terminal effector T cells T_EMRA_ subset, as demonstrated by Romero et al. [28] and Koch et al. [29]. The data presented in our study indicates a statistically significant reduction in the T cell population among patients with both severe and mild conditions. The CD4 subsets, namely Th EMRA-Tim-3+ and Th Navie-Tim-3+, are the subject of discussion. The group of patients with severe COVID-19 exhibited a notable reduction compared to the cohort with mild symptoms. The study found a statistically significant increase in the CD8 subsets Tc Naïve-Tim3+, Tc EM-Tim-3+, and Tc CM-Tim-3+ in severe patients compared to mild patients.

In contrast, the naive T cell is primarily responsible for activation and proliferation, as Okada et al. [28] and Koch et al. [30] noted. The CD27+CD28+ subset of T cells, commonly referred to as naïve T cells, play a critical role in mounting an effective immune response against novel viral infections such as COVID-19 or in response to vaccination. The study found no statistically significant variation in the population of CD4 and CD8 subsets of cells between day 1 and day 4 among COVID-19 patients with mild symptoms. Severe COVID-19 patients exhibited a notable elevation in Th-Naive-Tim3+, Th-EM-Tim3+, and Th-CM-Tim3+.

A phenotypic transition of CD8+ T cells from T effector/T_EM_ cells to T_EMRA_ cells has been observed, with a notable prevalence of T_EMRA_ cells among SARS-CoV-2-specific CD8+ T cells as reported in studies [31,32]. T lymphocytes differentiate into either CD4+ or CD8+ T cells, which exit the thymus and migrate to secondary lymphoid organs. These cells play a crucial role in the adaptive cellular immune response, aiding in eradicating infections [33-34].

De Biasi et al. compared the immune system of hospitalized patients exhibiting mild to moderate disease and revealed a reduced count of total CD4+ and CD8+ T cells, along with their respective naive and T memory subsets, in the patient group [11,35].

A relatively small sample size remains the main limitation of this study. In addition, combining this standardized data with a more in-depth investigation of SARS-CoV-2-specific immune responses could provide a complete picture of immune responses to COVID-19. Additional research is required, preferably with follow-up and immune response evaluation after vaccination.

## Conclusions

This study revealed that lymphocytes, monocytes and eosinophils were significantly reduced in COVID-19 patients. A significant decrease of CD4 and CD8 cells in severe patients was observed. T cell populations and CD8 subset help to differentiate the mild and severe COVID-19 patients. Monitoring T cells, especially CD8 subset changes, has important implications for diagnosing and treating mild and severe patients. The higher percentages of Th and Tc memory cells in the mild group of recovered individuals may serve as a prognostic indicator and reinforces the potential involvement of T cells in COVID-19 and in the development of immunological memory subsequent to recovery.

## Figures and Tables

**Figure 9:**
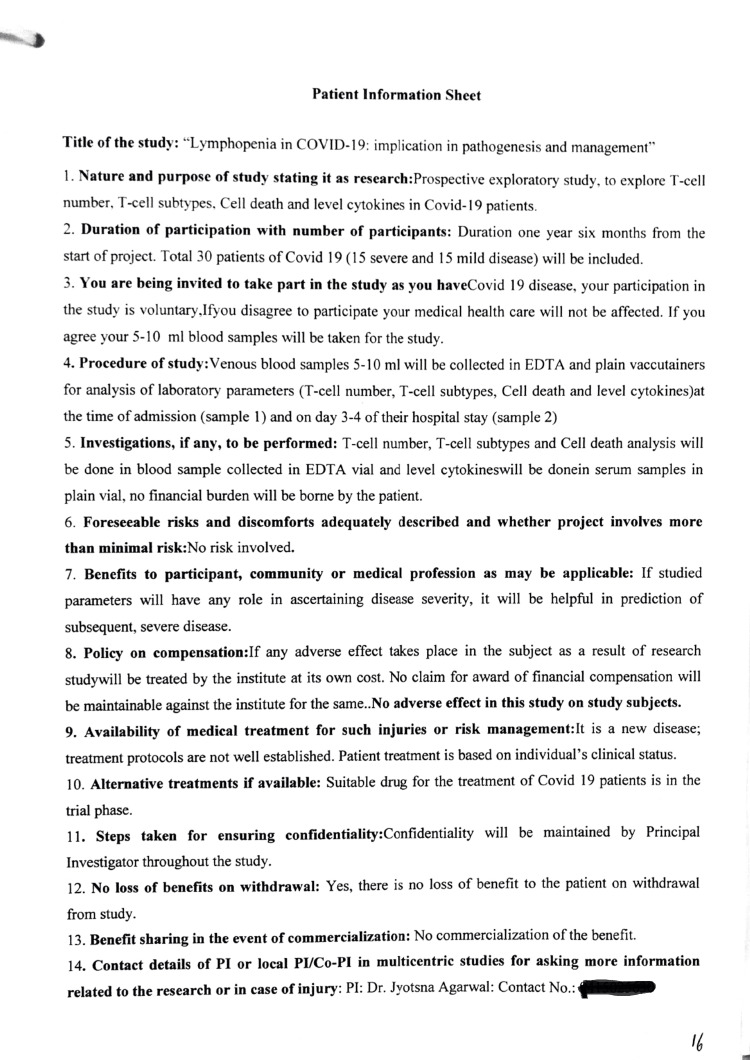
Patient information sheet for participation in the study

**Figure 10:**
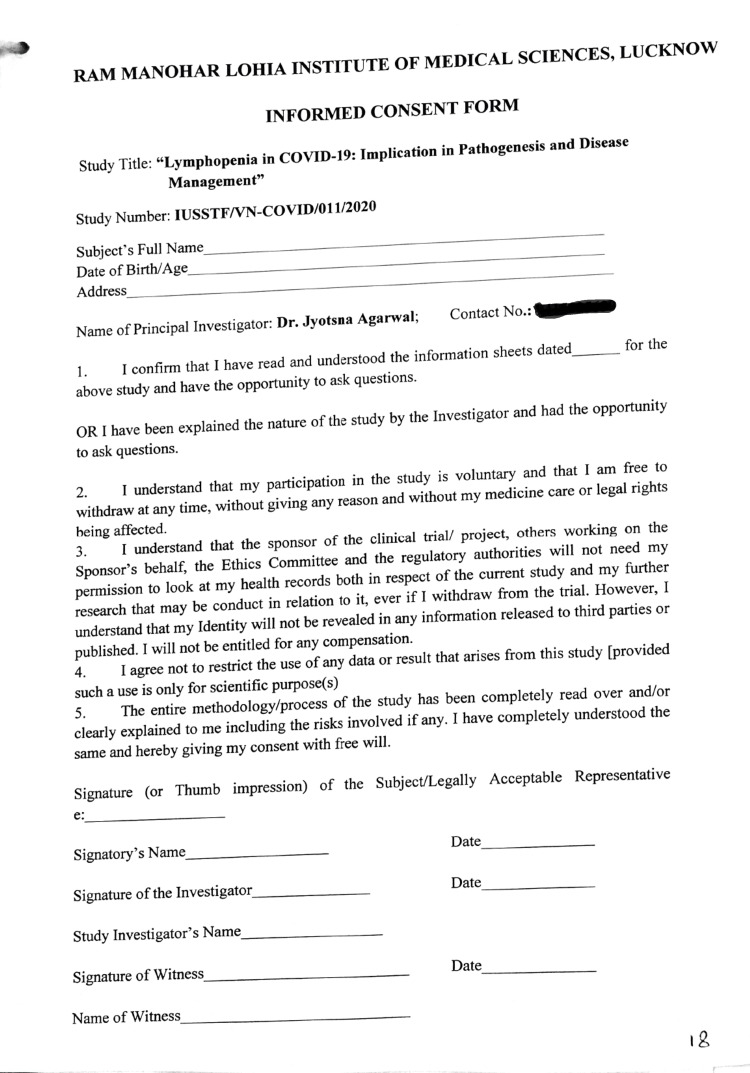
Consent form in English

**Figure 11:**
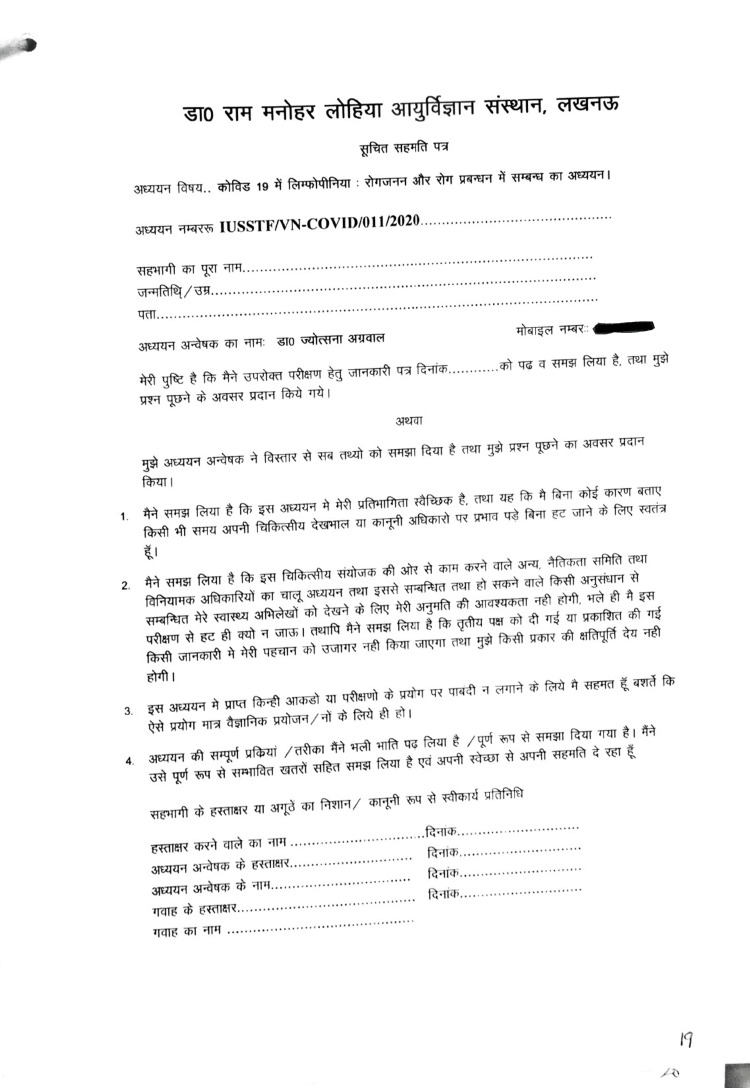
Consent form in Hindi
